# Mechanism of functional interaction between potassium channel Kv1.3 and sodium channel NavBeta1 subunit

**DOI:** 10.1038/srep45310

**Published:** 2017-03-28

**Authors:** Tomoya Kubota, Ana M. Correa, Francisco Bezanilla

**Affiliations:** 1 Department of Biochemistry and Molecular Biology, The University of Chicago, 929 E. 57th street, Chicago, IL 60637, USA.

## Abstract

The voltage-gated potassium channel subfamily A member 3 (Kv1.3) dominantly expresses on T cells and neurons. Recently, the interaction between Kv1.3 and NavBeta1 subunits has been explored through ionic current measurements, but the molecular mechanism has not been elucidated yet. We explored the functional interaction between Kv1.3 and NavBeta1 through gating current measurements using the Cut-open Oocyte Voltage Clamp (COVC) technique. We showed that the N-terminal 1–52 sequence of hKv1.3 disrupts the channel expression on the *Xenopus* oocyte membrane, suggesting a potential role as regulator of hKv1.3 expression in neurons and lymphocytes. Our gating currents measurements showed that NavBeta1 interacts with the voltage sensing domain (VSD) of Kv1.3 through W172 in the transmembrane segment and modifies the gating operation. The comparison between G-V and Q-V with/without NavBeta1 indicates that NavBeta1 may strengthen the coupling between hKv1.3-VSD movement and pore opening, inducing the modification of kinetics in ionic activation and deactivation.

Voltage gated ion channels (VGICs) play crucial roles in the propagation of electrical signals in excitable cells. In contrast, several VGICs have been also known to express in non-excitable cells and participate in keeping cell homeostasis^1^,^2^. The voltage-gated potassium channel from subfamily A, member 3 (Kv1.3), encoded in the *KCNA3* gene is one of the Shaker-related channels which was cloned from a non-excitable T cell^3^,^4^,^5^,^6^,^7^,^8^,^9^. Many reports have indicated that Kv1.3 in T cells is associated with several diseases including autoimmune diseases, inflammatory diseases and obesity^10^,^11^,^12^,^13^,^14^,^15^,^16^,^17^,^18^,^19^,^20^,^21^. On the other hand, the voltage-gated sodium channel (Nav) Beta 1, encoded in the *SCN1B* gene, is known as an auxiliary subunit that modulates kinetics and expression of Nav channel^22^,^23^,^24^. However, several studies have shown that NavBeta1 also participates in brain development and cell signaling^25^,^26^,^27^,^28^. These kind of ‘non-canonical’ functions of VGICs and their subunits have become the object of attention in several research areas including immunology and cancer research.

Although NavBeta1 was originally identified as “a subunit for Nav channels”, interaction with Kv channels has been reported. Mainly using heterologous expression systems^29^,^30^,^31^, it has been shown that K^+^ ionic currents of several Kv channels including Kv1.1, Kv1.2, Kv1.3, Kv1.6, Kv4.2, Kv4.3 and Kv7.2, are modified by the co-expression of NavBeta1. Moreover, the Navbeta1 has been previously co-purified with Kv4.2 from the native mouse brain^30^. There is one report showing that knocking down *SCN1B* by siRNA in neonatal rat ventricular cardiomyocytes decreased both Na^+^ currents and transient outward K^+^ currents^32^. Even though there is solid evidence that Kv channels are modulated functionally by NavBeta1, the molecular mechanisms have not been elucidated.

To address possible mechanisms involved, we explored the interaction between Kv1.3 and NavBeta1 through gating current measurements using the Cut-open Oocyte Voltage Clamp (COVC) technique. We found that the N-terminus of human Kv1.3 (hKv1.3) is associated with expression efficiency of the protein in *Xenopus* oocytes. Our data indicated that NavBeta1 shifted the voltage-dependence of gating current activation (Q-V) of Kv1.3 in the depolarizing direction and slowed their kinetics. Using a chimera between rat NavBeta1 and rat myelin protein zero (P_0_), we identified that the tryptophan residue at position 172 in the transmembrane segment of NavBeta1 plays an important role in the interaction between hKv1.3 and rNavBeta1. Comparing the voltage-dependence of ionic activation (G-V) and Q-V with/without NavBeta1, the results suggest that NavBeta1 strengthens the coupling between hKv1.3-VSD movement and pore opening.

## Results

### Optimizing hKv1.3 clone for Gating current measurements

**Figure 1:**
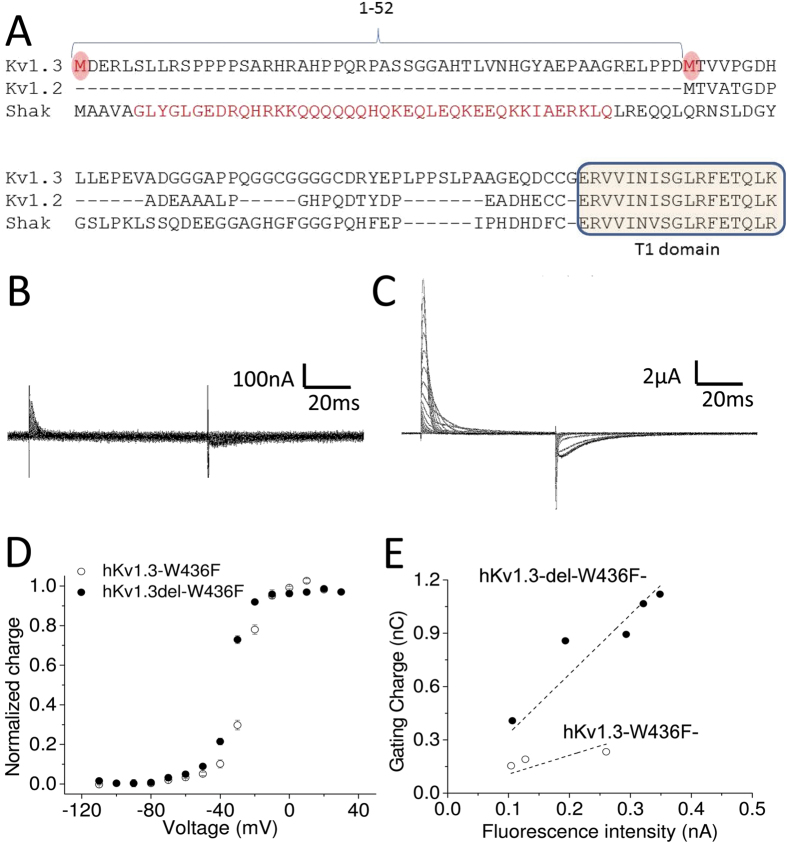
N-terminus of Kv1.3 prevents the expression on *Xenopus* oocytes membrane. (**A**) Amino acid sequence alignments between human Kv1.3 (Kv1.3), human Kv1.2 (Kv1.2) and Shaker K channel (Shak). Two potential start codons are highlighted in red circles in Kv1.3. The N-terminal sequences in Shaker K channel responsible for N-type inactivation is highlighted in red in Shak. The T1 domain is highlighted in light yellow. (**B**,**C**) Representative gating currents from hKv1.3-W436F (**B**) and from hKv1.3-del-W436F (**C**). (**D**) Normalized charge as a function of voltage (Q-V) for hKv1.3-W436F (open circles, n = 4) and for hKv1.3-del-W436F (filled circles, n = 8). Error bars indicate standard error of mean (SEM). (**E**) The total charge as a function of fluorescent intensity for hKv1.3-W436F-mCherry (open circles) and for hKv1.3-del-W436F-mCherry (filled circles). These data were obtained from the same batch of *Xenopus* oocytes. Each data set was fitted to a linear function (dotted lines). The R^2^ values were 0.98 for hKv1.3-del-W436F-mCherry and 0.91 for hKv1.3-W436F-mCherry. The linear slopes were 3.4 ± 0.2 for hKv1.3-del-W436F-mCherry and 1.1 ± 0.2 for hKv1.3-W436F-mCherry.

To generate a non-conducting version of the clone for gating current measurements, a point mutation, W436F (equivalent to W434F mutation in Shaker K channel^33^), was introduced into hKv1.3. Interestingly, hKv1.3 mRNA in the database shows two potential start codons, M1 and M53. We generated two hKv1.3 clones, the full length hKv1.3-W436F and an N-terminal deletion mutant, hKv1.3-del-W436F, in which residues M1 through D52 were deleted (Fig. 1A). Representative gating currents from these clones are shown in Fig. 1B and C. The total charge of hKv1.3-W436F gating currents is significantly smaller (0.2 ± 0.05 nC, n = 4) than that of hKv1.3-del-W436F gating currents (8.1 ± 1.2 nC, n = 8), indicating that the N-terminus 1–52 sequence influences protein synthesis or expression in *Xenopus* oocytes membrane, as further evidenced by the channel’s voltage-dependence and saturation voltage remaining similar (Fig. 1D). To investigate further the role of the N-terminal 1–52 sequence, we generated two additional constructs by inserting a fluorescent protein, mCherry, between V502 and G503 in the C-terminus, which is 18 amino acids away from the end of S6: hKv1.3-del-W436F-mCherry and hKv1.3-W436F-mCherry. The total gating charges and total fluorescence collected from the same cells are shown plotted in Fig. 1E. Here, the total fluorescence represents the amount of protein synthesized and located underneath of oocytes membrane, including proteins both inserted and non-inserted in the membrane. As shown in Fig. 1E, the charge/fluorescence ratio was smaller in hKv1.3-W436F-mCherry (3.4 ± 0.2 in hKv1.3-del-W436F-mCherry *vs* 1.1 ± 0.2 in hKv1.3-W436F-mCherry), indicating that hKv1.3-W436F-mCherry was synthesized but generated smaller gating currents presumably because not all the protein was inserted in the membrane. This result suggests that both M1 and M53 work as start codons for protein synthesis, but that the N-terminal 1–52 sequence disrupts insertion of hKv1.3 protein into the membrane of *Xenopus* oocytes. Because deletion of the N-terminus boosts the expression of hKv1.3 enough to detect robust gating currents, we used the hKv1.3-del construct for all measurements hereafter.

### The effect of NavBeta1 subunit on Kv1.3 gating current is similar in human and rat

**Figure 2:**
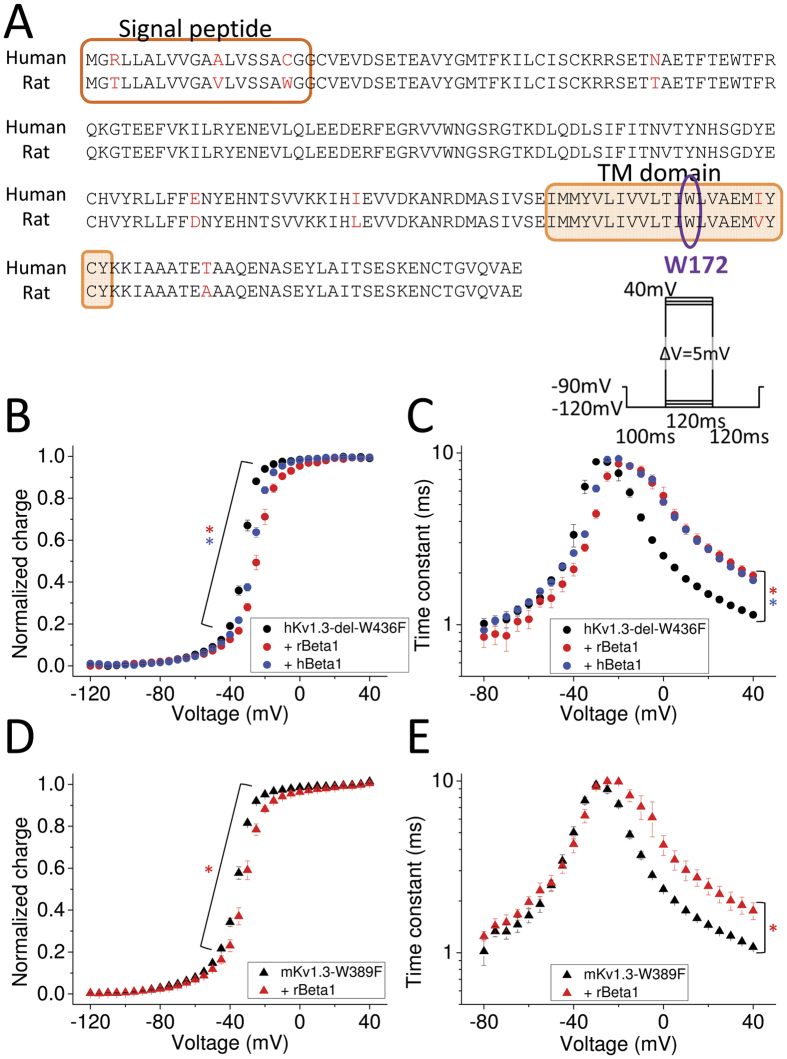
NavBeta effect on Kv1.3 gating currents in rodents and human. (**A**) Amino acid sequence alignment of human NavBeta1 (hNavBeta1) and rat NavBeta1 (rNavBeta1). Residues that differ between them are highlighted in red. Transmembrane segment is indicated with an orange rectangle. W172 is indicated in purple circle (See also Fig. 5). (**B**,**C**) Q-V relationship (**B**) and time constants of on-gating currents as a function of voltage (Tau-V) (**C**) for hKv1.3-del-W436F alone (black circles, n = 8), and co-injected with rNavBeta1 (red circles, n = 5) or with hNavBeta1 (blue circles, n = 5). Red stars (*) indicate statistical significance (*p*-value < 0.05) of the difference between hKv1.3-del-W436F alone (black circles) and with rNavBeta1 (red circles) at voltages ranging from −50 mV to +10 mV in Q-V (**B**), and at voltages ranging from −15 mV to +40 mV for Tau-V (**C**). Blue stars (*) indicate statistical significance (*p*-value < 0.05) between hKv1.3-del-W436F alone (black circles) and with hNavBeta1 (blue circles) at voltages ranging from −45 mV to −10 mV for Q-V (**B**), and at voltages ranging from −15 mV to +40 mV for Tau-V (**C**). (**D**,**E**) Q-V relationship (**D**) and Tau-V (**E**) for mouse Kv1.3 (mKv1.3)-W389F alone (black triangles, n = 4) and co-injected with rNavBeta1 (red triangles, n = 4). Red stars (*) indicate statistical significance (*p*-value < 0.05) of the difference between mKv1.3-W389F alone (black) and with rNavBeta1 (red) for voltages ranging from −55 mV to −10 mV in Q-V (**D**), and at voltages ranging from 0 mV to +40 mV in Tau-V (**E**). The pulse protocol for gating current measurements is depicted in the upper right corner in **C**). Error bars indicate SEM.

We measured hKv1.3 gating currents with and without rat NavBeta1 subunit (rNavBeta1). The rNavBeta1 subunit shifted the charge-voltage relationship (Q-V) in the depolarizing direction and made the gating current time constants slower in the depolarized range of voltage (Fig. 2B and C). As shown in Fig. 2A, human and rat NavBeta1 differ somewhat in their amino acid sequence. To examine whether the effect of rNavBeta1 on hKv1.3 was specific or not, we measured the following combinations as well: hKv1.3-del-W436F with hNavBeta1 and the murine non-conducting mKv1.3-W389F with rNavBeta1 (Fig. 2B–E). Although the strength of effects had some variation, all NavBeta1 subunits shifted the Q-V in the depolarizing direction and made the time constants slower at positive potentials.

### Comparison of NavBeta1 effect on Kv1.3 gating currents between conductive Kv1.3 channel and the non-conductive Kv1.3-W436F channel

**Figure 3:**
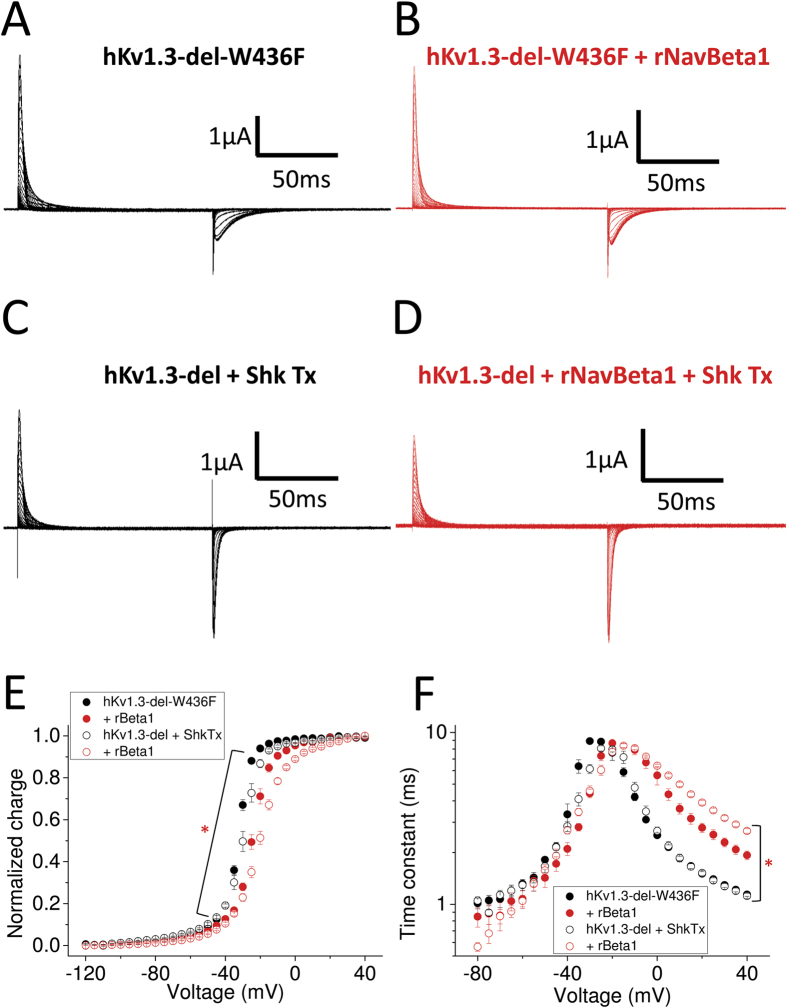
Gating currents from hKv1.3-del-W436F and conductive hKv1.3-del with/without rNavBeta1. (**A**–**D**) Representative gating currents from hKv1.3-del-W436F alone (**A**), co-injected with rNavBeta1 (**B**), from conductive hKv1.3-del alone while blocked by Shk Tx (**C**) and co-injected with rNavBeta1 (**D**). (**E**,**F**) The Q-V relationship (**E**) and Tau-V relationship (**F**) for hKv1.3-del-W436F alone (filled black circles, n = 8) and co-injected with rNavBeta1 (filled red circles, n = 5), and for conductive hKv1.3-del blocked by ShkTx (opened black circles, n = 4) and co-injected with rNavBeta1 (opened red circles, n = 6). Error bars indicate SEM. Red star (*) indicates statistical significance (*p*-value < 0.05) of the difference between conductive hKv1.3-del blocked by ShkTx (opened black circles) and with rNavBeta1 (opened red circles) from −115 mV to +15 mV in Q-V (**E**), and from −10 mV to +40 mV in Tau-V (**F**).

All measurements so far were done using Kv1.3 clones with a point mutation, W436F in hKv1.3-del and W389F in mKv1.3, corresponding in both cases to the W434F mutation in Shaker K channel, which renders the channel non-conducting^33^. To address whether the rNavBeta1 effect on hKv1.3-del-W436F might be due to the W-F substitution, we measured gating currents from the hKv1.3-del conductive clone blocked with Shk-Toxin, which is a potent Kv1.3 pore blocker^34^. The effect of rNavBeta1 was the same on both hKv1.3-del with and without W436F, indicating that the effect of rNavBeta1 on hKv1.3-del-W436F is not due to W436F mutation. In addition, note that gating currents from hKv1.3-del-W436F showed slower off-gating kinetics than those from the conductive hKv1.3-del blocked with Shk-Toxin, which is consistent with similar observations in Shaker K channels and in Kv1.2 without the W-F mutation^35^ (Fig. 3A–D).

### P_0_-NavBeta1 chimera slows down Kv1.3 gating current

**Figure 4:**
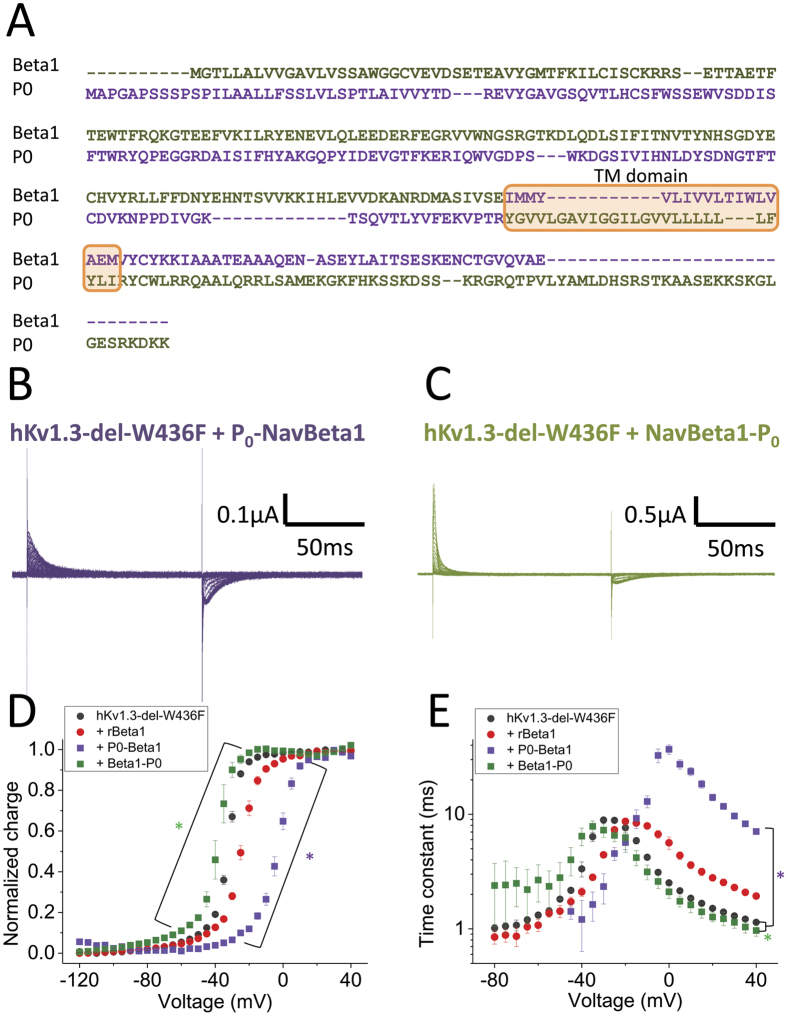
The effect of chimeras of rNavBeta1 and P_0_ on hKv1.3 gating current. (**A**) Amino acid sequence alignment between rNavBeta1 and myelin protein zero (P_0_), a protein with high homology to rNavBeta1. Transmembrane segment is highlighted by orange rectangle. The sequence for NavBeta1-P_0_ chimera is indicated in green and that for P_0_-NavBeta1 in purple. (**B**,**C**) Representative gating currents from hKv1.3-del-W436F co-injected with P_0_-NavBeta1 (**B**) or with NavBeta1-P_0_ (**C**). (**D**,**E**) The Q-V relationship (**D**) and Tau-V relationship (**E**) for hKv1.3-del-W436F alone (black circles, n = 8), co-injected with rNavBeta1 (red circles, n = 5), co-injected with P0-NavBeta1 (purple squares, n = 5) or co-injected with NavBeta1-P0 (green squares, n = 3). Error bars indicate SEM. Green stars (*) indicate statistical significance (*p*-value < 0.05) of the difference between hKv1.3-del-W436F alone (black circles) and with NavBeta1-P0 (green squares) from −95 mV to −10 mV in Q-V (**D**), and at −15, −10, +5, +20 and +40 mV in Tau-V (**E**). Purple stars (*) indicate statistical significance (*p*-value < 0.05) of the difference between hKv1.3-del-W436F alone (black circles) and with P0-NavBeta1 (purple squares) from −70 mV to +20 mV in Q-V (**D**), and from −40 mV to +40 mV except at −20 mV in Tau-V (**E**).

Results so far suggest that rNavBeta1 interacts with hKv1.3-del-W436F and modifies the gating operation. To investigate the region in rNavBeta1 interacting with hKv1.3-del-W436F, we used two chimeras, P_0_-NavBeta1 and NavBeta1-P_0_^36^. P_0_, the myelin protein zero, is a major glycoprotein of the myelin sheath and it is known to have high homology with rNavBeta1^37^. The P_0_-NavBeta1 construct has the extracellular portion of P_0_ and the transmembrane segment and cytosol portions of rNavBeta1 (purple in Fig. 4A). Conversely, the NavBeta1-P_0_ construct has the extracellular portion of rNavBeta1 and the transmembrane segment and cytosol portions of P_0_ (green in Fig. 4A). Gating currents of hKv1.3-del-W436F with P_0_-NavBeta1 were significantly slower than those of hKv1.3-del-W436F alone while NavBeta1-P_0_ showed the opposite effect on the gating of hKv1.3-del-W436F, which was the Q-V shift in the hyperpolarizing direction and speeding-up of the gating current time constants in the depolarized range of voltage (Fig. 4B–E). In addition, the gating currents of hKv1.3-del-W436F co-expressed with the whole P_0_ showed a small difference from those of hKv1.3-del-W436F alone (Suppl Fig. 1). These results indicate that the transmembrane segment or the cytosol portion of rNavBeta1 may function as a major interaction interface with Kv1.3.

### W172A mutation in NavBeta1 decreased its effect on Kv1.3

**Figure 5:**
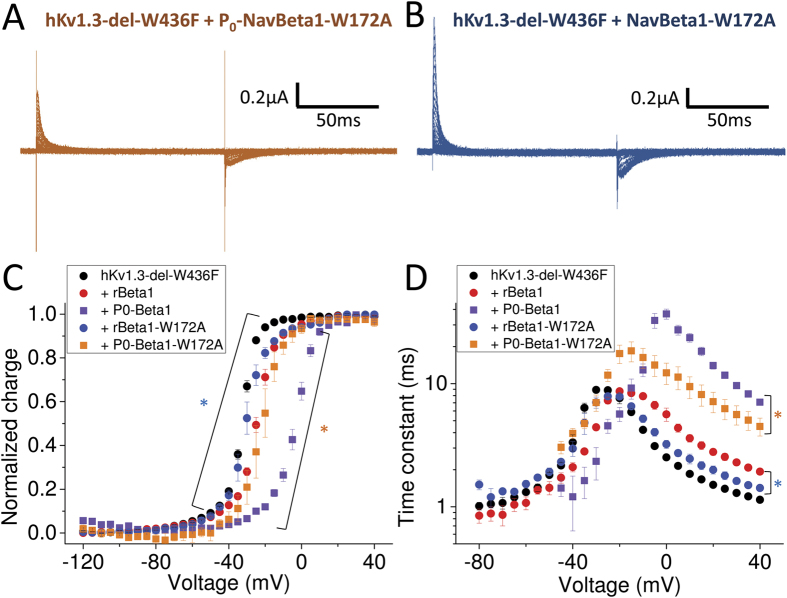
W172 in NavBeta1 transmembrane segment is part of an interaction interface with hKv1.3. (**A**,**B**) Representative gating currents from hKv1.3-del-W436F co-injected with P_0_-NavBeta1-W172A (**A**) or with NavBeta1-W172A (**B**). (**C**,**D**) The Q-V relationship (**C**) and Tau-V curve (**D**) for hKv1.3-del-W436F alone (black circles, n = 8), co-injected with rNavBeta1 (red circles, n = 5), co-injected with P0-NavBeta1 (purple squares, n = 5), co-injected with P0-NavBeta1-W172A (orange squares, n = 3) or co-injected with NavBeta1-W172A (blue circles, n = 3). Error bars indicate SEM. Orange stars (*) indicate statistical significance (*p*-value < 0.05) of the difference between hKv1.3-del-W436F with P0-NavBeta1 (purple squares) and with P0-NavBeta1-W172A (orange squares) from −30 mV to +5 mV in Q-V (**C**), and from −40 mV to +40 mV except at −10 mV in Tau-V (**D**). Blue stars (*) indicate statistical significance (*p*-value < 0.05) of the difference between hKv1.3-del-W436F with rNavBeta1 (red circles) and with NavBeta1-W172A (blue circles) from −45 mV to −25 mV except at −40 mV in Q-V (**C**), and from −15 mV to +40 mV in Tau-V (**D**).

In a previous report, based solely on homology modeling, Nguyen *et al*. (2012) proposed that tryptophan 172 (W172) in the rNavBeta1 transmembrane segment could act as an interaction site with Kv channels. To investigate whether W172 in rNavBeta1 is a key residue for modification of hKv1.3-del-W436F gating currents, we first introduced a W172A mutation into the P_0_-NavBeta1 chimera. Because this chimera prominently slows-down hKv1.3-del-W436F gating currents, it should be a good indicator of the subunit effect *via* W172A putative interaction. The gating current of hKv1.3-del-W436F with P_0_-NavBeta1-W172A was significantly faster than that with P_0_-NavBeta1 (Fig. 5A,C and D). Moreover, gating currents measured from hKv1.3-del-W436F with rNavBeta1-W172A showed a smaller shift and faster time constant than that with rNavBeta1 (Fig. 5B,C and D). Taken together, these results indicate that W172 in the transmembrane segment of rNavBeta1 plays an important role as an interacting site with hKv1.3 that modifies gating current operation at depolarized potentials.

### NavBeta1 effect on ionic current from homotetrameric Kv1.3

**Figure 6:**
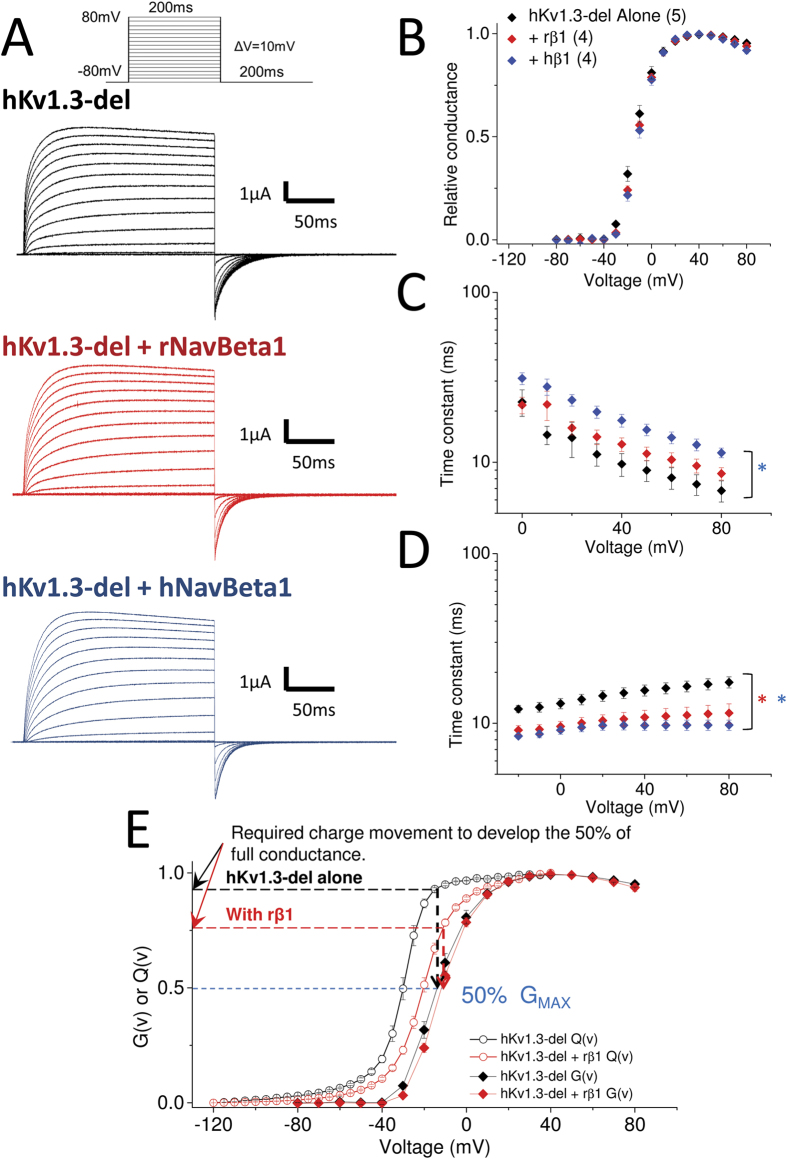
NavBeta1 effect on hKv1.3-del ionic currents. (**A**) Representative ionic currents from hKv1.3-del alone (black, n = 5), co-injected with rNavBeta1 (red, n = 4) or co-injected with hNavBeta1 (blue, n = 4). Pulse protocol is shown at the top of the panel. (**B**) Normalized conductance as a function of voltage (G-V) for hKv1.3-del alone (black), co-injected with rNavBeta1 (red) or with hNavBeta1 (blue). (**C**) Time constants of activation as a function of voltage for hKv1.3-del alone (black), co-injected with rNavBeta1 (red) or co-injected with hNavBeta1 (blue). Time constants were obtained as weighted average values from the fit of the rising face of the currents (highlighted in yellow in A) to two exponential functions. Blue star (*) indicates statistical significance (*p*-value < 0.05) of the difference between hKv1.3-del alone (black) and with hNavBeta1 (blue) from +10 mV to +80 mV except at +20 mV. (**D**) Deactivation time constants from the tail currents at –80 mV (highlighted in yellow at the end of the pulse in A) for hKv1.3-del alone (black), co-injected with rNavBeta1 (red) or co-injected with hNavBeta1 (blue). Time constants were obtained as weighted average values from the fit to two exponentials. Stars (*) indicate statistical significance (*p*-value < 0.05) of the difference between hKv1.3-del alone (black) and with rNavBeta1 (red) in red, and with hNavBeta1 (blue) in blue, from −20 mV to +80 mV. (**E**) Comparison between G-V and Q-V between hKv1.3 alone and when co-injected with rNavBeta1. To reach 50% of the maximum conductance (G_max_), hKv1.3 with rNavBeta1 requires less charge movement than hKv1.3 alone, indicating rNavBeta1 may strengthen the coupling between VSD movement and pore opening.

We recorded potassium ionic currents from hKv1.3 alone, with rNavBeta1 or with hNavBeta1 (Fig. 6A). The normalized ionic conductances (G-V) were identical, but both NavBeta1 made the kinetics of ionic activation slower and made the kinetics of tail currents faster at −80 mV (Fig. 6B–D). When Q-V and G-V of hKv1.3 with/without rNavBeta1 are superimposed, it becomes evident that hKv1.3 with rNavBeta1 requires less charge movement than hKv1.3 alone to get 50% maximum conductance (Fig. 6E). This result indicates that rNavBeta1 may strengthen the coupling between activation of the hKv1.3 voltage sensor (VSD) and the opening of the pore. These functional modifications on ionic currents are expected to influence the responsiveness of the cell upon stimulation.

## Discussion

In this study, we have shown that the N-terminal residues 1–52 of hKv1.3 disrupts expression of the protein on the *Xenopus* oocyte plasma membrane, suggesting a potential role as regulator of hKv1.3 expression in neurons and lymphocytes. Our gating current measurements showed that residue W172 in the transmembrane segment of NavBeta1 is important for the interaction of NavBeta1 with the Kv1.3-VSD that modifies gating kinetics. The comparison between G-V and Q-V with and without NavBeta1 indicates that NavBeta1 may strengthen the coupling between hKv1.3-VSD activation and channel pore opening. The functional alterations brought about by NavBeta1 modify the kinetics of ionic current activation and deactivation processes, which is expected to have physiological effects on excitable cells.

Kv1.3 protein is encoded in the *KCNA3* gene, composed of only one exon. When protein sequences of Kv1.3 are compared among several species in the database, human, primates, pig and fish have a long N-terminus that starts at an additional start codon (M1 in Fig. 1A) while rodents and frog have shorter N-termini (Suppl. Fig. 2). We showed that both M1 and M53 in hKv1.3 work as start codon, but that hKv1.3 starting from M53 (hKv1.3-del) expressed more efficiently in *Xenopus* oocytes. This result is consistent with the fact that mKv1.3 (which lacks the N-terminal sequence corresponding to M1-M52 in hKv1.3) expresses nicely and showed robust gating currents (Fig. 2D,E). It should be recalled that our results are from *Xenopus* oocytes expression system only. However, it is possible that the M1-D52 sequence may work as a regulator of hKv1.3 expression even in humans helping to create diversity in immunological and nervous systems. Kv1.3 channel is important to maintain the membrane potential of T cells and the expression pattern of Kv1.3 changes with activation and differentiation status. In human, primates and pig, the membrane potential of T cells rely mainly on Kv1.3^38^,^39^,^40^. However, the mouse T cell does not rely on Kv1.3 and express additional Kv channels^41^,^42^,^43^. Therefore, the difference in N-terminal moiety among species may promote immunological variety. In addition, there is one report showing that proton (HVCN1) channels in B cells have two potential start codons and there is different expression pattern between healthy controls and patients with chronic lymphocytic leukemia (CLL): the full-length HVCN1 channel expresses more in healthy controls while the shorter HVCN1 channel, which lacks 20 amino acids in the N-terminus, expresses more in CLL patients^44^. This evidence supports the possibility that the expression pattern of Kv1.3 isoform, the full-length *vs.* Kv1.3-del, may be linked to diversity of the immunological system.

Although Kv1.3 is known to express in several immune cells including T cells, macrophages and microglia, it also expresses highly in neuronal cells^45^,^46^. In addition, several pieces of evidence have shown that Kv1.3 forms heterotetramers with members of other Kv1 families including Kv1.1, Kv1.2, Kv1.4 and Kv1.5^47^,^48^,^49^. On the other hand, it has been shown that NavBeta1 expresses not only in excitable cells, including neurons and myocytes, but also in non-excitable cells, including astrocytes and glial cells^50^. While there is no clear evidence showing that NavBeta1 expresses in T cells, the possibility should not be excluded because NavBeta1 mRNA has been detected in several hematopoietic cells^45^,^51^. The effects we demonstrate here, however, most likely occur in excitable cells, i.e. neurons. Moreover, the interaction observed may also be involved in neuronal developmental processes because NavBeta1 has been proposed to be important during development^25^,^26^,^27^.

Our results provide experimental evidence showing the importance of the transmembrane segment of NavBeta1 as an interacting interface with Kv1.3. This is interesting from a biophysical point of view, but many factors remain unsolved. First, the transmembrane segment of NavBeta1 may not be the only interacting region with Kv1.3. Because the extracellular “Ig-like” part of NavBeta1 has been shown to interact with Navs, it is quite possible that it also participates in the interaction with Kvs. A previous report by Nguyan HM *et al*. showed that the extracellular “Ig” part of NavBeta1 modified the ionic current kinetics of mKv1.3^31^. Another possibility is that the extracellular moiety may be necessary to form an appropriate conformation, a “clamp-like shape”, of NavBeta1 to facilitate the interaction through the transmembrane segment. Second, the stoichiometry between Kv1.3 and NavBeta1 is unknown. Although Kv1.3 forms a tetramer, only one NavBeta1 may be sufficient to interact with one Kv channel because the extracellular part of NavBeta1 is extremely large amounting to 73% of the whole protein. Finally, the interacting interface between Kv1.3 and NavBeta1 may be different from that between Nav1.4 and NavBeta1. Nav1.4 ionic currents when co-expressed with NavBeta1-W172A showed acceleration of fast inactivation similar to that seen with the wildtype NavBeta1 (Suppl. Fig. 3). This result indicates that W172 is not relevant for the interaction between Nav1.4 and NavBeta1.

Comparison between the voltage dependence of the gating and ionic currents of hKv1.3-del, with and without rNavBeta1, suggested that rNavBeta1 strengthens the coupling between activation of the hKv1.3 VSD and the opening of the pore (Fig. 6). A previous study showed that the voltage-dependence of the ionic conductance of mKv1.3 did not change when co-expressed with rNavBeta1, which is similar to our results with hKv1.3^31^. The gating current data from mKv1.3-W389F showed a slight shift of the voltage-dependence in the direction of depolarization. Taken together, the ionic current data from the previous study and gating current data from our study, indicate that rNavBeta1 may strengthen the coupling between the VSD and the pore opening also in mKv1.3 even though there are several differences in the secondary structure between hKv1.3 and mKv1.3.

Our results are based on homotetrameric Kv1.3. In the case that Kv1.3 forms heterotetramers with other Kv channel monomers, which are also capable of interacting with NavBeta1^29^,^30^,^31^, one would expect a large number of combinations of Kvs and NavBeta1. Because the variation can be influenced by the expression profile in each cell type and also by the developmental stages, the interaction between Kv and NavBeta1 can be relevant in particular physiological situations. Although VGICs are mainly expected to be of significance in excitable cells and NavBeta1 is considered a Nav subunit, our data supports non-canonical roles of VGICs and their subunits that may have physiological roles. While more studies directed to address the specific physiological role of these findings in immunological processes are necessary, our data provides a starting point.

## Methods

### Expression of hKv1.3, mKv1.3 and Navβ1s in Xenopus oocytes

cDNA encoding human Kv1.3 (hKv1.3) was a generous gift from Dr. Dirk J. Snyders. cDNA encoding rat P0, rat P0-NavBeta1 chimera amd NavBeta1-P0 chimera were kindly provided by Dr. Lori L. Isom. These and rat NavBeta1 (rNavBeta1) were cloned in pBSTA vectors containing the Kozak sequence of *Xenopus* laevis beta-globin gene 5-gccgccatgg. Mutations and insertions, including the human NavBeta1 carrying the signal peptide sequence of rNavBeta1 and hKv1.3 with mCherry, were made using Quick-change (Agilent, Santa Clara, CA). The mouse Kv1.3 (mKv1.3) in pSP64T vector was kindly provided by Dr. Hai M. Nguyen, Dr. Heike Wulff and Dr. K. George Chandy. Plasmids were transcribed using *in vitro* transcription kits, mMESSAGE T7 or mMESSAGE SP6 (Ambion, Austin, TX). Freshly isolated oocytes were injected with a total 50 ng of cRNA and kept in SOS incubation solution (96 mM NaCl, 2 mM KCl, 1.8 mM CaCl_2_, 1 mM MgCl_2_, 10 mM HEPES, pH 7.4) for 1–5 days at 18 °C. In case of co-injection of Kv1.3 and NavBeta1 derivatives, a 1:1 in weight ratio was applied.

### Electrophysiology

Ionic and gating currents were recorded 1–4 days after injection using the cut-open oocyte voltage-clamp (COVC) technique as described^52^. To record K^+^ currents, recording solutions were prepared as follows: extracellular solution: 108 mM n-methylglucamine (NMG)-methylsulfonate (MS), 12 mM K-MS, 2 mM Ca-MS, 10 mM HEPES, pH 7.4, and internal solution: 120 mM K-MS, 2 mM EGTA, 10 mM HEPES, pH 7.4. Linear leak and membrane capacitive currents were subtracted using a P/6 protocol from a subtracting holding potential of −110 mV. Membrane was held at −80 mV for 20 sec between pulse cycles to avoid cumulative inactivation. We collected data from cells with 1–10 μA of peak ionic current at +80 mV. The pulse protocol for I-V data is shown in Fig. 6A. For gating current recording solutions were as follows: extracellular solution: 115 mM NMG-MS, 2 mM Ca-MS, 10 mM HEPES, pH 7.4, and internal solution: 115 mM NMG-MS, 2 mM EGTA, 10 mM HEPES, pH 7.4. Linear leak and membrane capacitive currents were subtracted using a P/6 protocol from a subtracting holding potential of +20 mV. As the internal pipette solutions, 3 M KCl and 3 M CsCl were used for ionic and gating current measurements, respectively. We applied 20 sec resting periods at −90 mV between each pulse cycle. All experiments were done at 20 °C. For gating current measurements using the hKv1.3-del conductive construct, the ShK toxin (ShKTx) was diluted to 1 μM in the extracellular solution containing 0.1% BSA (Sigma-Aldrich, St. Louis, MO).

### Cut-open oocyte epifluorescence recording

The experimental set-up is composed of both cut-open oocytes voltage-clamp (COVC) and optical set-ups^53^. Filters and dichroic mirror were chosen as follows: HQ535/50 as the excitation filter (Chroma Technology Corp), 575DRLP as the dichroic mirror (Omega Optical) and E610LP (Chroma Technology Corp) as the emission filter.

### Data analysis

Sample numbers (n) for each measurement were stated in the Figure legend. Errors indicate standard error of means (SEM). Statistical significance was determined in cases where *p*-values were less than 0.05 by unpaired t-test.

A reversal potential (V_rev_), −58 mV, was obtained from the K^+^ ion gradient between external and internal solutions. Conductance was calculated using the equation: G(V) = I/(V − V_rev_), where G (V) is the conductance, I is the normalized ionic current amplitude and V is the applied voltage. Time constants were obtained from two exponential fits performed with an in-house program, Analysis. In the results, weighted average values of the obtained time constants were plotted.

## Additional Information

**How to cite this article:** Kubota, T. *et al*. Mechanism of functional interaction between potassium channel Kv1.3 and sodium channel NavBeta1 subunit. *Sci. Rep.* **7**, 45310; doi: 10.1038/srep45310 (2017).

**Publisher's note:** Springer Nature remains neutral with regard to jurisdictional claims in published maps and institutional affiliations.

## Supplementary Material

Supplementary Information
