# Supplementary material for: Mechanism of functional interaction between potassium channel Kv1.3 and sodium channel NavBeta1 subunit
Source: Sci Rep. 2017 Mar 28;7:45310. doi: 10.1038/srep45310 (PMC5368567; doi:10.1038/srep45310)
Supplement: Supplementary Information [file srep45310-s1.pdf]

Supplementary Information for

Mechanism of functional interaction between potassium channel Kv1.3 and sodium channel NavBeta1 subunit

Tomoya Kubota1, Ana M. Correa and Francisco Bezanilla.

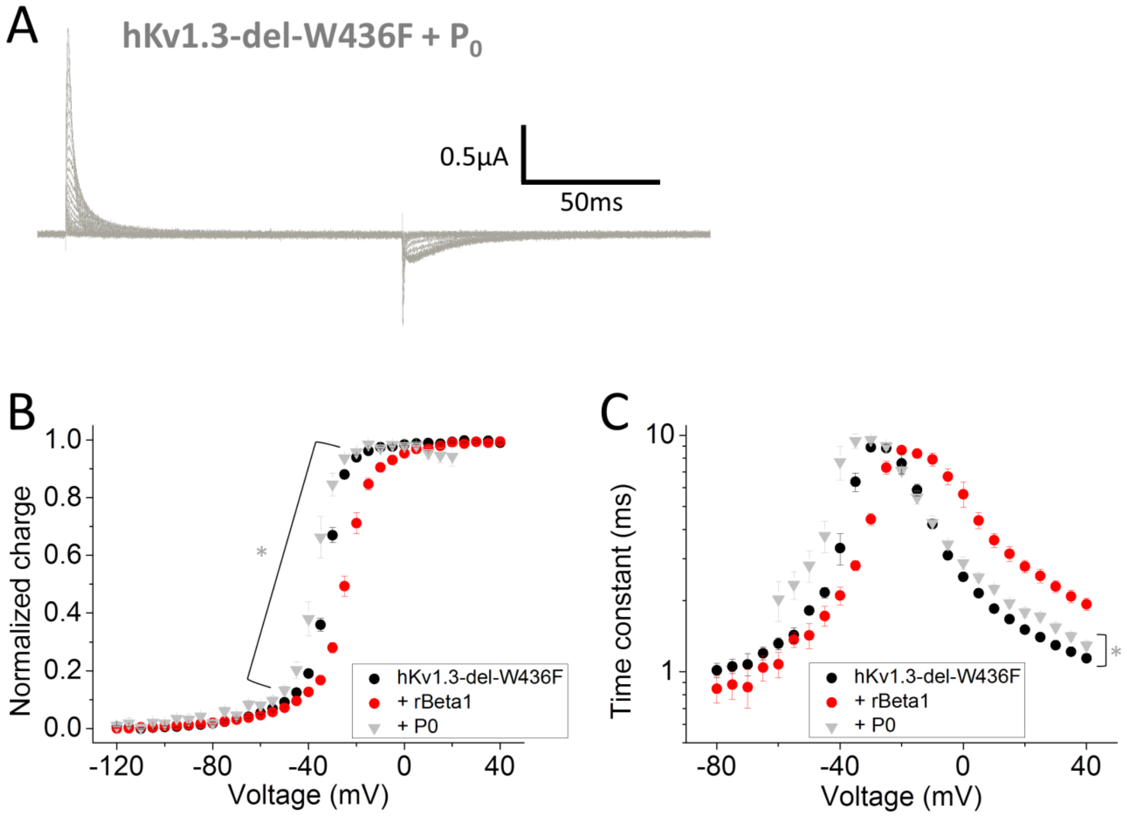

Suppl. Fig 1. Effect of Myelin Basic Protein P_0 on hKv1.3-del-W436F gating currents

A) Representative gating currents from hKv1.3-del-W436F co-expressed with P_0. **B**)-**C**) The Q-V relationship (**B**) and Tau-V curve (**C**) for hKv1.3-del-W436F alone (black circles, n=8), co-injected with rNavBeta1 (red circles, n=5) or with P0 (gray inverted triangles, n=4). Error bars indicate SEM. Gray stars (*) indicate statistical significance (p-value < 0.05) of the difference between hKv1.3-del-W436F (black circles) and with P0 (gray inverted triangles) from -50 mV to -25 mV in Q-V (**C**), and from 0 mV to +40 mV in Tau-V (**D**).

A

```
P22001 Human     MDERLSLLRSPP-PPSARHRAHPPQRPASSG-----------GAHTLVNHGYAEPAAGRE
G3QEG2 Gorilla   MDEHLSLLRSPP-PPSARHRAHPPQRPASSG-----------GAHTLVNPGYAEPAAGRE
F6ZSH4 Macaque   MDEHLSLLRSPP-PPSARHRAHPAQRPASSG-----------GAHTLVNPGYAEPAAGPE
F1S626 Pig       MDEHLSLLRSPPPPPSTRHRAHPPQHPASRGGGGGGGGGGGGDAHTLVNPGYAEPAAGPE
P15384 Rat       ------------------------------------------------------------
P16390 Mouse     ------------------------------------------------------------
Q90YY3 Xenopus   ------------------------------------------------------------
H2TUB9 Fugu      MDDHLSLLQSPPPPSVTKAR-----------------------GDNLVNHGYTDTEA---


P22001 Human     LPPDMTVVPGDHLLEPEVADG-GGAPPQGGC----GGGGCDRYEPLPPSLPAAGEQDCCG
G3QEG2 Gorilla   LPPNMTVVPGDHMLEPEVADG-GGAPPQGGC----GGGGCDRYEPLPPSLPAAGEQDCCG
F6ZSH4 Macaque   LPPDMTVVPGDHLLEPEVADG-GGAPPQGGC----GGGGCDRYEPLPPSLPAAGEQDCCG
F1S626 Pig       LPPDMTVVPGDHLLEPEAADG-GGDPPQGGCG---GGGGCDRYEPLPPALPAAGEQDCCG
P15384 Rat       ----MTVVPGDHLLEPEAAGGGGGDPPQGGCVSG---GGCDRYEPLPPALPAAGEQDCCG
P16390 Mouse     ----MTVVPGDHLLEPEAAGGGGGDPPQGGCGSGGGGGGCDRYEPLPPALPAAGEQDCCG
Q90YY3 Xenopus   ----MTVVACDNILEEAAALPG--------------HHSSEAYE---------QEDHECC
H2TUB9 Fugu      --DVMTVVACDNMLEESAALPG--------------NHSLDRYE----------PDHECC
```

Suppl. Fig 2. KCNA3 N-terminus alignment among species

A) Potential start codons, M1 and M53 in human are highlighted in red. Human, primates, pig and Fugu have longer N-terminus while rodents and *Xenopus* don’t.

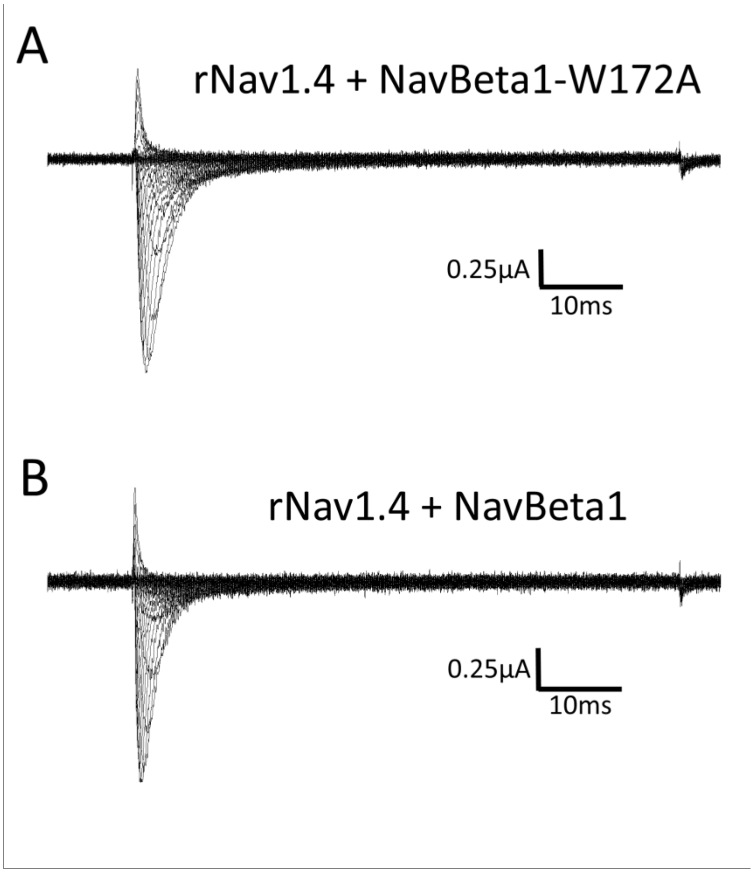

Suppl. Fig 3. W172A effect on Nav ionic currents

Sodium ionic currents from rat skeletal muscle Nav channel (Nav1.4) co-injected with NavBeta1-W172A (**A**) or with NavBeta1 (**B**). NavBeta1-W172A showed acceleration of fast inactivation similar to NavBeta1.
